# Alzheimer’s Disease and Cognitive Decline in Patients with Cardiovascular Diseases Along the Heart-Brain Axis

**DOI:** 10.3233/JAD-231096

**Published:** 2024-04-02

**Authors:** Calvin Trieu, Argonde C. van Harten, Anna E. Leeuwis, Lieza G. Exalto, Astrid M. Hooghiemstra, Inge M.W. Verberk, Cor P. Allaart, Hans-Peter Brunner-La Rocca, L. Jaap Kappelle, Robert J. van Oostenbrugge, Geert-Jan Biessels, Charlotte E. Teunissen, Wiesje M. van der Flier, on behalf of Heart-Brain Connection Consortium

**Affiliations:** a Alzheimer Center Amsterdam, Neurology, Vrije Universiteit Amsterdam, Amsterdam University Medical Center (Amsterdam UMC), Amsterdam, The Netherlands; b Amsterdam Neuroscience, Program Neurodegeneration, Amsterdam, The Netherlands; c Department of Laboratory Medicine, Neurochemistry Laboratory, Amsterdam Neuroscience, Program Neurodegeneration, Vrije Universiteit Amsterdam, Amsterdam University Medical Center (Amsterdam UMC), Amsterdam, The Netherlands; d Department of Neurology, UMC Utrecht Brain Center, University Medical Center Utrecht, Utrecht, The Netherlands; e Brain Research Center, Zwolle, The Netherlands; f Julius Clinical, Zeist, The Netherlands; g Department of Cardiology, Institute for Cardiovascular Research, Vrije Universiteit Amsterdam, Amsterdam University Medical Center (Amsterdam UMC), Amsterdam, The Netherlands; h Department of Cardiology, Maastricht University Medical Center, Maastricht, The Netherlands; i Department of Neurology, Maastricht University Medical Center, Maastricht, The Netherlands

**Keywords:** Alzheimer’s disease, carotid stenosis, cognitive dysfunction, heart failure, vascular dementia

## Abstract

**Background:**

We hypothesize that Alzheimer’s disease (AD)-related pathology may accelerate cognitive decline in patients with cardiovascular diseases.

**Objective:**

To investigate the association between blood-based biomarkers of AD, astrocyte activation, and neurodegeneration and cognitive decline.

**Methods:**

From the multi-center Heart-Brain study, we included 412 patients with heart failure, carotid occlusive disease or vascular cognitive impairment (age:68.6±9.0) and 128 reference participants (65.7±7.5). Baseline amyloid-β_42/40_ (Aβ_42/40_), phosphorylated-tau181 (pTau181), glial fibrillary acidic protein (GFAP), and neurofilament light (NfL) were determined using SiMoA (Quanterix). Memory, attention, language, and executive functioning were evaluated (follow-up:2.1±0.3 years). We applied linear mixed models with terms for biomarker, time and biomarker^*^time interactions, adjusted for age, sex, education, and site, to assess associations between biomarkers and cognitive decline.

**Results:**

Among patients, Aβ_42/40_ was not associated with cognitive performance at baseline. However, lower Aβ_42/40_ was associated with steeper decline in global cognition (β±SE:0.04±0.02). Higher pTau181 was associated with worse baseline performance on global cognition (–0.14±0.04) and memory (–0.31±0.09) and with steeper decline in global cognition (–0.07±0.02), memory (–0.09±0.04), attention (–0.05±0.02), and language (–0.10±0.03). Higher GFAP was associated with worse baseline performance on global cognition (–0.22±0.05), memory (–0.43±0.10), attention (–0.14±0.06), language (–0.15±0.05), and executive functioning (–0.15±0.05) and steeper decline in global cognition (–0.05±0.01). Higher NfL was associated with worse baseline performance on global cognition (–0.16±0.04), memory (–0.28±0.09), attention (–0.20±0.06), and executive functioning (-0.10±0.04), but was not associated with performance over time. In reference participants, no associations were found.

**Conclusions:**

Our findings suggest that blood-based biomarkers of AD-related pathology predict cognitive decline in patients with cardiovascular diseases.

## INTRODUCTION

Cardiovascular diseases (CVD) and Alzheimer’s disease (AD) are two of the most common causes of dementia. Growing evidence suggest a link between these two diseases. Previous research has shown that CVD and cerebral small vessel disease (CSVD) are associated with an increased risk of AD and lower the threshold at which AD symptoms manifest [1, 2]. Furthermore, both CVD and AD share several common risk factors, which increases the development of both diseases, including age, smoking, hypertension, hypercholesterolemia, diabetes mellitus, and apolipoprotein E (*APOE*) *ɛ*4 carriership [3, 4]. In the Heart-Brain study, we investigate the heart-brain axis, underscoring the impact of hemodynamic factors and (cardio-)vascular diseases on cognitive functioning. Considering the shared underlying pathology related to blood flow and its predisposed risk for vascular brain injury, three patient groups represent examples of hemodynamic dysfunction at different levels of the heart-brain axis: heart failure (HF), carotid occlusive disease (COD) and vascular cognitive impairment (VCI) [5]. In a previous study, we demonstrated that patients with COD and HF had a higher prevalence of cognitive impairment than community dwelling older adults, noting cognitive impairment in 18% of HF patients, and 36% of COD patients [6]. Initially, we hypothesized that cognitive impairment in this population was induced by chronic cerebral hypoperfusion, in HF caused by systematic hypoperfusion resulted from the low cardiac output, in COD caused by reduced cerebral blood flow resulting from narrowing of the lumen of the carotid artery, and in VCI caused by impaired cerebral circulation. Contrary to our expectation, cerebral blood flow did not explain differences in cognitive performance in these patients [7]. With the connection between AD and CSVD in mind, an alternative explanation could be that AD contributes to cognitive impairment and cognitive decline over time in these patients.

The pathological process of AD is thought to begin two decades before the onset of dementia. Classical hallmarks of AD pathology include amyloid plaques and neurofibrillary tangles, neuronal loss, and excessive activation of microglia and astrocytes [8]. In recent years, major advances have been made in the field of blood-based biomarkers for identifying AD related pathology. These biomarkers, including the ratio of amyloid-beta 42 (Aβ_1–42_) and amyloid-beta 40 (Aβ_1–40_), phosphorylated tau 181 (pTau181), glial fibrillary acidic protein (GFAP), and neurofilament light chain (NfL) [9–12], facilitate the assessment of (comorbid) neurodegenerative processes associated with AD. Specifically, a lower Aβ_42/40_ ratio reflects Aβ deposition, while higher pTau181 levels reflect the burden of both Aβ deposition and tau pathology [11, 13]. Higher GFAP levels reflect astrocyte activation, which is considered a part of the neuro-inflammatory response against Aβ plaques [14, 15]. Higher NfL levels reflect neuro-axonal damage and the degeneration of large myelinated axons [16, 17].

We hypothesize that AD-related pathology is associated with cognitive impairment and cognitive decline over time in patients with cardiovascular diseases along the heart brain-axis. We therefore aimed to investigate whether blood-based biomarkers for AD (including biomarkers for astrocyte activation and neurodegeneration in general) are associated with cognitive performance at baseline and cognitive decline over time in patients with HF, COD, and VCI and compare this to the results in healthy reference participants.

## METHODS

### Participants

We included participants from the Heart-Brain study [5]. The Heart-Brain study is a prospective study in which patients were recruited from the memory, neurology, and cardiology outpatient clinics at four academic hospitals: Amsterdam University Medical Centers (Amsterdam UMC), location VUmc, Leiden University Medical Center (LUMC), University Medical Center Utrecht (UMCU), and Maastricht University Medical Center + (MUMC+). The main inclusion criteria were: 1) age 50 years or older, 2) able to undergo cognitive testing and MRI, 3) independence in daily life, 4) diagnosis of HF, COD, or VCI (only for patient groups) [18]. The main exclusion criteria were: 1) diagnosis of a neurodegenerative disease other than VCI or AD, 2) diagnosis of another neurologic or psychiatric disorder, 3) diagnosis of atrial fibrillation at the moment of inclusion. We enrolled patients with HF, according to the European Cardiology Society guidelines, irrespective of left ventricular ejection fraction and coronary artery disease with a stable clinical stable situation for at least 6 months before enrollment [18]. For COD, we enrolled patients with significant stenosis (>80%) or occlusion of the internal carotid artery as assessed with MR angiography. For VCI, we included patients with cognitive complaints (Clinical Dementia Rating (CDR):≤1 and Mini-Mental State Examination (MMSE):≥20), in combination with moderate to severe vascular brain injury or mild vascular brain injury and multiple vascular risk factors. Reference participants were recruited through advertisements and among spouses of patients, with no additional inclusion criteria beyond the main criteria. A more elaborate description of the inclusion criteria for each patient group are specified in a previous publication of the Heart-Brain Connection Consortium [5]. All patients with HF, COD, VCI, and reference participants from September 2014 to November 2018 (Heart-Brain Connection data version 3, 01-01-2020), for whom blood-based biomarker measurements were available, were included in the current study. The Heart-Brain study included a total of 566 participants. We excluded 26 participants, because of missing data for blood-based biomarkers (*n* = 24), missing data for cognitive scores (*n* = 1), and outlying cognitive scores with more than three standard deviations from the mean (*n* = 1). This resulted in a sample of 540 participants, including 412 participants with cardiovascular diseases (HF (*n* = 153), COD (*n* = 105), VCI (*n* = 154)) and 128 reference participants (*n* = 128).

The baseline visits for all participants included assessment of: sociodemographic factors (age, sex, educational level, and social situation), current medication use, vascular risk factors (hypertension, diabetes, hypercholesterolemia, and smoking) and medical history (medical, neurological, cardiovascular, and family history). It also included physical examination (length, height, body mass index, and blood pressure), neuropsychiatric questionnaires (MMSE, CDR, Geriatric Depression Scale-15 (GDS)), a screening laboratory test (creatinine) and a brain MRI (CSVD score) [19–22]. Medical history of previous transient ischemic attack, cerebrovascular accident, myocardial infarction, renal impairment, and vascular risk factors were assessed by self-reported medical history questionnaires. The MMSE is a cognitive screening tool that assesses cognitive functioning, with a total score between 0 and 30, where lower scores indicate worse cognitive performance. The CDR is an assessment instrument used to quantify the severity of symptoms of dementia, ranging from 0–3, where higher scores indicate worse severity of dementia. The GDS is a screening tool comprising 15 items that measures depressive symptoms, with a total score between 0 and 15, where higher scores indicate greater severity of depressive symptoms. Creatinine is a marker reflecting kidney function, typically ranging from 45–100*μ*mol/L in adult males and 45 to 80*μ*mol/L in adult females, where elevated creatinine levels indicate a reduced kidney function. The CSVD score, ranging from 0–4, is determined by visual assessment by a neuroradiologist on T1-weighted, fluid attenuation inversion recovery, and susceptibility-weighted images. Each point in the CSVD score indicates the presence of one specific marker, including≥1 lacunar infarct, microbleeds, moderate to severe vascular spaces in basal ganglia, and a Fazekas score of 3.

### Standard protocol approvals, registrations, and patient consents

All participants provided written informed consent before participation. This study obtained approval from the Medical Ethics Review Committee of the LUMC and conforms with the declaration of Helsinki (version 2013).

### Cognitive functioning

#### Baseline assessment

Cognitive performance was assessed using a standardized neuropsychological test battery, covering global cognition and four major cognitive domains. For memory we used the Rey Auditory Verbal Learning Test (RAVLT) and Visual Association Test (VAT) [23–25]. For language we used the animal verbal fluency tests (VFT) and VAT naming [26]. For attention-psychomotor speed we used the Trail Making Test (TMT) – part A, the Letter-Digit Substitution Test (LDST), the Stroop Color-Word Test (SCWT) – Card I and II and the Forward Digit Span [27–30]. For executive functioning we used the TMT – part B/part A, Backward Digit Span and the SCWT interference score [27, 29, 30]. Raw test scores from the neuropsychological test were standardized into z-scores, using reference participants as reference group, and combined into cognitive domains. Global cognition scores were constructed by calculating the mean z-scores across all cognitive domains. A more elaborate description of how the z-scores were constructed is provided elsewhere [5].

#### Follow-up assessment

Participants underwent neuropsychological assessment approximately two years after the baseline assessment (mean follow-up time: 2.1±0.34 years). Patients with VCI underwent an additional neuropsychological investigation 1 year after the baseline assessment. One hundred sixty-seven participants did not undergo neuropsychological testing at the 2-year follow-up due to several reasons: death (*n* = 20), illness (*n* = 7), nursing home admission (*n* = 8), lost to follow-up (*n* = 22), and other reasons (*n* = 110).

### Blood-based biomarkers

Ethylenediaminetetraacetic acid (EDTA) plasma was collected by venipuncture. After centrifugation at 1800×*g* for 10 min at room temperature, the plasma was stored at –80°C in aliquots of 0.5 mL in Sarsedt polypropylene tubes. Prior to use, samples were shortly thawed at room temperature and centrifuged at 10,000×*g* for 10 min, to prevent sample debris from interfering with the measurements. Subsequently, plasma levels of Aβ_42_, Aβ_40_, GFAP, and NfL were measured with the Simoa™ Neurology 4-plex E Kit (Quanterix, Billerica, USA) and plasma levels of pTau181 with the Simoa™ pTau181 V2 Kit (Quanterix), on the Simoa HDX analyzer (Quanterix). Analyses were performed in duplicates for both the Neurology 4-plex E kit and the pTau181 V2 kit. Analyses were performed according to manufacturer’s instructions with 1 : 4 automated on-board sample dilution. The average intra-assay coefficient of variation (CV) were 2.4% for Aβ_1–40_, 2.6% for Aβ_1–42_, 5.1% for GFAP, 4.3% for NfL, and 7.4% for pTau181. The average inter-assay CV were 6.5% for Aβ_1–40_, 5.9% for Aβ_1–42_, 6.7% for GFAP and 5.7% for NfL, measured using three quality control samples over 7 runs. For pTau181, the average inter-assay CV was 7.8%, measured with of two quality control samples over 6 runs.

### Statistical analysis

All statistical analyses were performed using SPSS version 26.0 for Windows (SPSS Inc., Chicago, USA) and RStudio version 2021.09.2 for MacOS (RStudio, Inc., Boston, MA, USA). Before analyses we log-transformed concentrations of three of the blood-based biomarkers (pTau181, GFAP, and NfL) to normalize the distributions. All blood-based biomarker concentrations were then converted into z-scores for comparability of effect sizes. Analyses of variance (ANOVA) and Pearson *χ*^2^ tests were used to compare demographic characteristics between all groups. Analysis of covariance (ANCOVA) was performed to compare plasma concentrations of blood-based biomarkers between groups, adjusted for age, sex, and hospital site. We used linear mixed models (LMM) to estimate baseline cognitive performance and longitudinal change in cognition in the total group and stratified by diagnostic group. The models included follow-up time in years as predictor and compound z scores for memory, attention/psychomotor speed, language, executive functioning, and global cognition as outcome measures in separate models. Next, we analyzed associations between standardized baseline blood-based biomarker concentrations and cognitive performance and cognitive decline using LMM. We included terms for biomarker, time and biomarker * time interactions in separate models for each biomarker and each cognitive domain. LMM was performed for the total group of patients along the heart-brain axis and reference participants separately. In a secondary analysis we stratified our analysis by diagnostic group, because the associations between AD and cognition may differ between the different diseases. Analyses were adjusted for age, sex, hospital site and education. For these LMM analyses, we used the false discovery rate (FDR) procedure to correct for multiple testing for each cognitive domains, *q*-values less than 0.05 after FDR correction (*q* < 0.05_FDR_) were considered significant [31]. In addition, we performed multiple sensitivity analyses: 1) excluding 12 patients within the VCI group who fulfilled the clinical criteria for AD dementia at baseline; 2) including the adjustment for GDS as an additional covariate; 3) including the adjustment for CVSD score as an additional covariate.

For illustrative purposes we divided all blood-based biomarkers into tertiles and performed additional linear mixed models, using terms for blood-based biomarker in tertiles, time and biomarker in tertiles * time as predictor and cognitive performance as outcome measure. Using intercepts and slopes of these models we constructed graphs to visualize the effects of blood-based biomarkers on cognitive decline over time.

## RESULTS

### Demographic characteristics

Demographic characteristics of the 540 participants are summarized in Table 1. At baseline, patients with HF, COD, and VCI scored lower on all cognitive domains than reference participants (Table 1). On average, cognitive performance did not change over time across all groups, with the exception of a decline in language in patients with VCI (β±SE –0.09±0.04, *p* < 0.05), and a small improvement in performance in the memory domain in patients with HF (β±SE 0.10±0.04, *p* < 0.05) and reference participants (β±SE 0.11±0.03, *p* < 0.001), and in the domain of executive functioning in patients with COD (β±SE 0.08±0.03, *p* < 0.05; Supplementary Table 1).

**Table 1:** Demographic characteristics

	HF	COD	VCI	Reference participants	Total
	*n*=153	*n*=105	*n*=154	*n*=128	*n*=540
Demographic and vascular risk factors^a^
Age, mean (SD)	70.1 (9.7)^‡§^	66.1 (8.0)^†^	68.8 (8.6)^*^	65.7 (7.5)^¶∘^	67.9 (8.7)
Female, *n* (%)	50 (32.7)^*^	25 (23.8)^∘§^	58 (37.7)^‡^	60 (46.9)^†#^	193 (35.7)
MMSE, mean (SD)	28.5 (1.3)^†#^	27.8 (2.1)^†^	27.4 (2.7)^¶§^	28.8 (1.2)^#^^∧^	28.1 (2.0)
CDR, median (IQR)	0 (0–0)	0 (0–0.5)	0.5 (0–0.5)	0 (0–0)	0 (0.0–0.5)
GDS, mean (SD)	2.1 (2.5)^∘§^	2.4 (2.4)^§^	2.9 (2.7)^†§^	0.9 (1.3)^¶#^^∧^	2.1 (2.4)
Education, median (IQR)	5 (4–6)^*^	5 (4–6)^*^	5 (4–6)	6 (5–6)^†‡^	5 (4–6)
Systolic BP, mean (SD)	134.7 (19.2)^# ∘*^	152.3 (21.8)^¶∘*^	142.0 (22.0)^†‡^	143.1 (19.2)^†‡^	142.2 (21.3)
Diastolic BP, mean (SD)	76.2 (11.4)^# ∘§^	82.4 (12.2)^¶^	80.9 (11.1)^†^	81.9 (10.2)^¶^	80.1 (11.4)
BMI, mean (SD)	27.5 (4.6)^*^	27.7 (3.9)^*^	26.6 (4.5)	26.1 (3.8)^†‡^	26.9 (4.3)
Creatinine in *μ*mol/L, mean (SD)	102.1 (42.0)^#^^∧^^§^	82.2 (32.1)^¶^	86.7 (23.6)^¶^	79.2 (29.4)^¶^	88.4 (33.8)
Hypertension, *n* (%)	81 (53.3)	80 (76.2)	108 (70.1)	33 (25.8)	302 (56.0)
Hypercholesterolemia, *n* (%)	71 (47.0)	90 (85.7)	102 (67.1)	38 (29.7)	301 (56.2)
Diabetes mellitus, *n* (%)	27 (17.6)	31 (29.5)	17 (11.0)	3 (2.3)	78 (14.4)
Renal impairment, *n* (%)	42 (27.5)	10 (9.5)	20 (13.0)	5 (3.9)	77 (14.3)
CSVD, mean (SD)	0.84 (1.01)^∧^^*^	1.15 (0.87)^∘§^	1.59 (1.29)^¶‡§^	0.45 (0.64)^†#^^∧^	1.01 (1.08)
CVA, *n* (%)	9 (5.9)	55 (52.4)	62 (40.3)	0 (0.0)	126 (23.3)
TIA, *n* (%)	16 (10.5)	77 (73.3)	36 (23.5)	6 (4.7)	135 (25.0)
Smoking, *n* (%)	22 (14.4)	30 (28.6)	28 (18.2)	9 (7.0)	89 (16.5)
Myocardial infarction, *n* (%)	81 (52.9)	15 (14.3)	18 (11.7)	4 (3.1)	118 (21.9)
Blood-based biomarkers^b^
Aβ_42/40_ ratio, mean (SD)	0.067 (0.014)	0.067 (0.013)	0.064 (0.012)	0.067 (0.015)	0.066 (0.013)
pTau181 in pg/mL, mean (SD)	2.0 (1.2)	1.7 (1.2)	2.1 (1.2)	1.7 (1.0)	1.9 (1.2)
GFAP in pg/mL, mean (SD)	113.9 (63.5)	94.7 (44.5)	127.9 (74.9)^†§^	91.3 (46.1)^∧^	108.8 (61.9)
NfL in pg/mL, mean (SD)	25.1 (18.2)^*^	27.8 (37.3)^*^	26.8 (21.4)^§^	16.5 (7.2)^†‡^^∧^	24.1 (22.9)
Cognitive performance^c^ (baseline z-score)
Global cognition, β (SE)	–0.41 (0.05)^§^	–0.52 (0.06)^§^	–0.89 (0.10)^§^	0.00 (0.05)	–0.48 (0.04)^§^
Memory, β (SE)	–0.48 (0.09)^§^	–0.57 (0.11)^§^	–1.44 (0.20)^§^	–0.01 (0.06)	–0.67 (0.07)^§^
Attention/psychomotor speed, β (SE)	–0.52 (0.07)^§^	–0.85 (0.10)^§^	–0.90 (0.11)^§^	0.01 (0.07)	–0.57 (0.05)^§^
Language, β (SE)	–0.38 (0.06)^§^	–0.34 (0.05)^§^	–0.65 (0.09)^§^	0.00 (0.06)	–0.37 (0.04)^§^
Executive functioning, β (SE)	–0.24 (0.06)^§^	–0.33 (0.07)^§^	–0.57 (0.08)^§^	0.01 (0.06)	–0.30 (0.04)^§^

BMI, body mass index; BP, blood pressure; CDR, Clinical Dementia Rating; COD, carotid occlusive disease; CSVD, cerebral small vessel disease; CVA, cerebrovascular accident; GDS, Geriatric Depression Scale; HF, heart failure; IQR, interquartile range; MMSE, Mini-Mental State Examination; TIA, transient ischemic attack; VCI, vascular cognitive impairment. ^a^Demographic differences between groups were calculated with the analyses of variance (ANOVA) or Pearson *χ*^2^ test as appropriate. ^b^Raw values of the plasma biomarkers are presented in the table. However, pTau181, GFAP, and NfL were log-transformed prior to analyses. Differences in plasma concentrations between groups were calculated using analysis of covariance (ANCOVA), adjusted for age, sex, and hospital site. Post-hoc analyses were performed, when between groups differences were significant in ANCOVA, to explore the differences between the included groups. Lower Aβ_42/40_ ratio indicate a more abnormal level, whereas it is inversed for pTau181, GFAP, and NfL, in which higher concentrations indicated a more abnormal level. ^c^Linear mixed model included terms for *time* in separate models for each cognitive domain. The estimates (β±SE) of the intercept (estimated baseline z score) represent standardized baseline cognitive performance. Analyses were adjusted for age, sex, hospital site, and education. *p*-values represent a significant difference with reference participants. ^*^p < 0.05 compared to reference participants; ^†^*p* < 0.05 compared to HF; ^‡^*p* < 0.05 compared to COD; *p* < 0.05 compared to VCI; ^§^*p* < 0.001 compared to reference participants; ^¶^*p* < 0.001 compared to HF; ^#^*p* < 0.001 compared to COD; ^∧^*p* < 0.001 compared to VCI.

### Blood-based biomarker concentrations

Table 1 shows the blood-based biomarker concentrations by diagnostic group. GFAP and NfL were higher in the total patient group than in the reference group. pTau181 was higher in the total patient group than in reference participants, but this effect became non-significant after adjusting for covariates. Aβ_42/40_ ratio did not differ between the total patient group and reference group. When we stratified the patient groups, we found that GFAP concentrations were higher in patients with VCI than in patients with HF and reference participants. NfL concentrations were higher in all patient groups compared to the reference group, but they did not differ between patient groups.

### Associations between blood-based biomarkers and cognitive performance

**Fig. 1:**
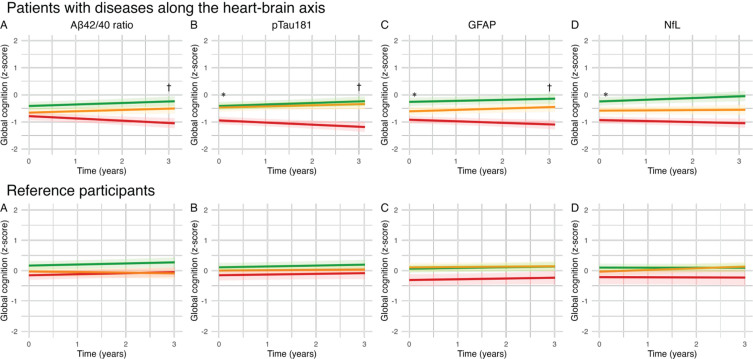
Effect of blood-based biomarker in tertiles on cognitive performance in global cognition (z-score): Lines represent βs for blood-based biomarkers in tertiles of the uncorrected model. A) The green line represents the high tertile of Aβ_42/40_ concentration, the orange line represents the medium tertile of Aβ_42/40_ concentration, the red line represents the low tertile of Aβ_42/40_ concentration (B–D). The green line represents the low tertile of pTau181/GFAP/NfL concentration, the orange line represents the medium tertile of pTau181/GFAP/NfL concentration, the red line represents the high tertile of pTau181/GFAP/NfL concentration. ^*^q < 0.05_FDR_ for baseline; ^†^*q* < 0.05_FDR_ for change over time.

**Fig. 2:**
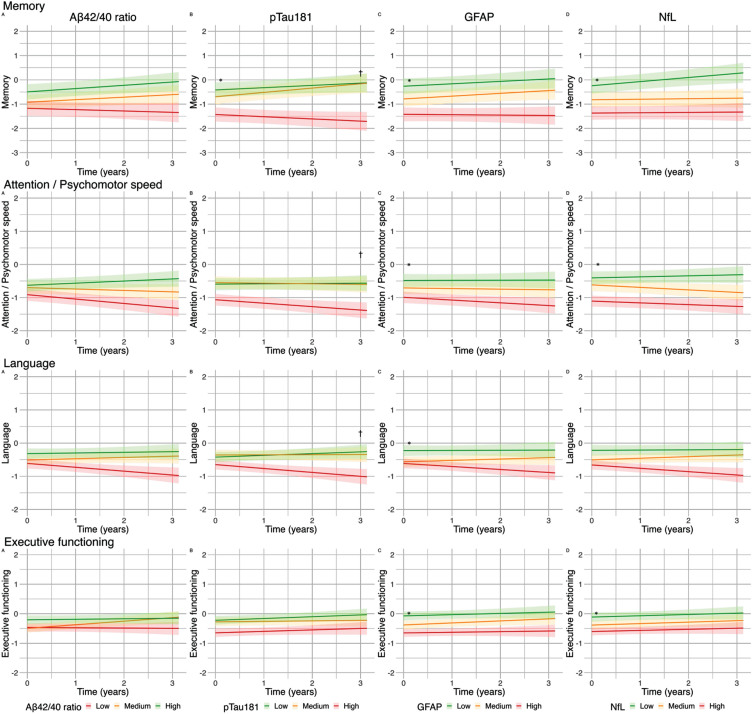
Effect of blood-based biomarker in tertiles on cognitive performance (z-scores) in patients with cardiovascular diseases along the heart-brain axis: Lines represent βs for blood-based biomarkers in tertiles of the uncorrected model. A) The green line represents the high tertile of Aβ_42/40_ concentration, the orange line represents the medium tertile of Aβ_42/40_ concentration, the red line represents the low tertile of Aβ_42/40_ concentration. B-D). The green line represents the low tertile of pTau181/GFAP/NfL concentration, the orange line represents the medium tertile of pTau181/GFAP/NfL concentration, the red line represents the high tertile of pTau181/GFAP/NfL concentration. The range of the graph in the memory domain is extended from “2 to –2” to “2 to –3”. ^*^q < 0.05_FDR_ for baseline; ^†^*q* < 0.05_FDR_ for change over time.

We used LMMs to investigate associations between blood-based biomarkers and cognitive performance, with separate models for each blood-based biomarker, and cognitive domain (Table 2, Figs. 1 and 2).

**Table 2:** Effect of blood-based biomarkers on baseline cognitive performance and cognitive performance over time

	Patient groups		HF		COD		VCI		Reference participants	
	Baseline z score β (SE)	Change over time β (SE)	Baseline z score β (SE)	Change over time β (SE)	Baseline z score β (SE)	Change over time β (SE)	Baseline z score β (SE)	Change over time β (SE)	Baseline z score β (SE)	Change over time β (SE)
Aβ_42/40_ ratio
GC	0.09 (0.04)	0.04 (0.02)^*^	0.04 (0.04)	–0.01 (0.02)	0.03 (0.06)	0.02 (0.02)	–0.02 (0.10)	0.11 (0.03)^**^	0.05 (0.04)	0.00 (0.01)
Memory	0.14 (0.09)	0.07 (0.03)	0.12 (0.09)	–0.03 (0.04)	0.05 (0.10)	0.02 (0.05)	–0.25 (0.22)	0.22 (0.07)^*^	0.08 (0.05)	0.02 (0.02)
Attention	0.07 (0.05)	0.04 (0.02)	0.04 (0.06)	0.00 (0.02)	0.02 (0.10)	0.05 (0.04)	–0.01 (0.12)	0.07 (0.04)	0.02 (0.06)	0.00 (0.01)
Language	0.10 (0.05)	0.05 (0.02)	0.04 (0.06)	0.01 (0.04)	–0.02 (0.06)	0.01 (0.03)	0.10 (0.11)	0.10 (0.05)	0.12 (0.06)	–0.03 (0.28)
EF	0.04 (0.04)	0.01 (0.02)	–0.05 (0.06)	–0.01 (0.03)	0.05 (0.07)	–0.01 (0.04)	0.06 (0.09)	0.05 (0.04)	0.00 (0.05)	0.02 (0.02)
pTau181
GC	–0.14 (0.04)^*^	–0.07 (0.02)^**^	–0.09 (0.05)	–0.02 (0.02)	–0.02 (0.06)	0.00 (0.02)	–0.17 (0.09)	–0.14 (0.03)^**^	0.02 (0.04)	0.00 (0.01)
Memory	–0.31 (0.09)^**^	–0.09 (0.04)^*^	–0.31 (0.10)^*^	0.03 (0.05)	–0.07 (0.11)	0.09 (0.05)	–0.27 (0.19)	–0.24 (0.06)^**^	0.03 (0.07)	0.01 (0.03)
Attention	–0.10 (0.06)	–0.05 (0.02)^*^	–0.07 (0.07)	0.00 (0.03)	0.01 (0.11)	–0.01 (0.05)	–0.15 (0.11)	–0.11 (0.04)^*^	–0.03 (0.07)	0.01 (0.02)
Language	–0.07 (0.05)	–0.10 (0.03)^**^	0.06 (0.07)	–0.05 (0.05)	–0.02 (0.06)	0.02 (0.03)	–0.14 (0.09)	–0.16 (0.04)^**^	0.04 (0.08)	–0.03 (0.03)
EF	–0.08 (0.04)	–0.04 (0.10)	–0.04 (0.07)	–0.04 (0.04)	0.00 (0.08)	–0.11 (0.04)^*^	–0.12 (0.08)	–0.01 (0.04)	0.03 (0.06)	0.02 (0.02)
GFAP
GC	–0.22 (0.05)^**^	–0.05 (0.01)^*^	–0.13 (0.05)^*^	–0.01 (0.02)	0.00 (0.00)	–0.01 (0.02)	–0.27 (0.10)^*^	–0.09 (0.03)^*^	–0.01 (0.05)	0.00 (0.01)
Memory	–0.43 (0.10)^**^	–0.05 (0.03)	–0.27 (0.11)^*^	0.01 (0.04)	0.02 (0.13)	0.00 (0.05)	–0.53 (0.21)^*^	–0.12 (0.06)	–0.07 (0.08)	0.02 (0.03)
Attention	–0.14 (0.06)^*^	–0.04 (0.02)	–0.07 (0.07)	0.00 (0.03)	0.04 (0.13)	0.01 (0.04)	–0.17 (0.12)	–0.11 (0.04)^*^	–0.01 (0.08)	–0.02 (0.02)
Language	–0.15 (0.05)^*^	–0.05 (0.02)	–0.06 (0.07)	–0.04 (0.04)	–0.04 (0.07)	–0.01 (0.03)	–0.16 (0.10)	–0.09 (0.04)	–0.01 (0.09)	–0.02 (0.03)
EF	–0.15 (0.05)^*^	–0.02 (0.02)	–0.12 (0.07)	–0.02 (0.03)	–0.01 (0.09)	–0.04 (0.04)	–0.19 (0.09)^*^	–0.01 (0.04)	0.03 (0.07)	–0.01 (0.02)
NfL
GC	–0.16 (0.04)^**^	–0.03 (0.02)	–0.08 (0.06)	–0.02 (0.02)	0.03 (0.05)	0.01 (0.02)	–0.32 (0.09)^**^	–0.05 (0.03)	0.00 (0.07)	0.01 (0.02)
Memory	–0.28 (0.09)^*^	–0.05 (0.03)	–0.14 (0.13)	–0.05 (0.05)	0.03 (0.09)	0.00 (0.04)	–0.54 (0.19)^*^	–0.06 (0.06)	0.15 (0.10)	0.04 (0.04)
Attention	–0.20 (0.06)^**^	–0.03 (0.02)	–0.18 (0.08)	0.00 (0.03)	0.01 (0.09)	0.03 (0.04)	–0.33 (0.10)^*^	–0.07 (0.04)	–0.09 (0.10)	–0.03 (0.02)
Language	–0.07 (0.05)	–0.04 (0.02)	0.10 (0.09)	0.01 (0.05)	0.05 (0.05)	–0.01 (0.02)	–0.19 (0.09)^*^	–0.07 (0.04)	0.06 (0.12)	0.02 (0.05)
EF	–0.10 (0.04)^*^	0.01 (0.02)	–0.09 (0.08)	0.00 (0.04)	0.03 (0.06)	0.02 (0.03)	–0.18 (0.08)^*^	0.02 (0.04)	–0.10 (0.09)	0.00 (0.03)

EF, executive functioning; GC, global cognition. Linear mixed model included terms for *biomarker*, *time*, and *biomarker* * *time interaction* in separate models for each biomarker and each cognitive domain. Analyses were adjusted for age, sex, hospital site and education. The estimates (β (SE)) with the term for *biomarker* (baseline z score) represent the association between the blood-based biomarker and baseline cognitive performance. Estimates with the term for *biomarker* * *time interaction* (change over time) represent the association between the blood-based biomarkers and annual change in cognitive performance over time. For Aβ_42/40_ ratio positive estimates indicate that lower concentrations of this biomarker are associated with worse cognitive performance. In contrast, for pTau181, GFAP and NFL, negative estimates indicate that higher concentrations of these biomarkers are associated with worse cognitive performance. The *p*-values are FDR-corrected and represent a significant association between the blood-based biomarker and cognitive performance of the designated cognitive domain at baseline (baseline z score) or over time (change over time). ^*^q_FDR_ < 0.05; ^**^q_FDR_ < 0.001.

In the total patient group, Aβ_42/40_ ratio was not associated with cognitive performance at baseline. However, lower Aβ_42/40_ ratio was associated with a steeper rate of decline in cognitive performance in global cognition (β±SE 0.04±0.02; *q* < 0.05). Higher pTau181 concentrations were associated with worse baseline performance on global cognition (β±SE –0.14±0.04; *q* < 0.01) and memory (β±SE –0.31±0.09; *q* < 0.001) and was associated with a steeper decline in global cognition (β±SE –0.07±0.02; *q* < 0.001), memory (β±SE –0.09±0.04; *q* < 0.05), attention and psychomotor speed (β±SE –0.05±0.02; *q* < 0.05), and language (β±SE –0.10±0.03; *q* < 0.001). Higher GFAP was associated with worse baseline performance on global cognition (β±SE –0.22±0.05; *q* < 0.001), memory (β±SE –0.43±0.10; *q* < 0.001), attention (β±SE –0.14±0.06; *q* < 0.01), language (β±SE –0.15±0.05; *q* < 0.01), and executive functioning (β±SE –0.15±0.05; *q* < 0.01). In addition, higher GFAP was associated with a steeper decline in global cognition (β±SE –0.05±0.01; *q* < 0.01). Higher NfL concentrations were associated with worse baseline performance on global cognition (β±SE –0.16±0.04; *q* < 0.001), memory (β±SE –0.28±0.09; *q* < 0.01), attention and psychomotor speed (β±SE –0.20±0.06; *q* < 0.001), and executive functioning (β±SE –0.10±0.04; *q* < 0.05), but was not associated with cognitive decline over time. In the reference participants, we found no associations between blood-based biomarkers and cognitive performance at baseline or cognitive decline in reference participants (Fig. 1). In sensitivity analyses excluding 12 patients fulfilling the clinical criteria for AD dementia, we observed a loss of significance in associations between pTau181 and multiple cognitive domains, as well as GFAP and attention at baseline and global cognition over time, and NfL and memory and executive functioning at baseline in the total patient group (Supplementary Table 2). In sensitivity analyses adjusting for GDS in the total patient group, we observed a loss of significance in the association between Aβ_42/40_ and global cognition over time, GFAP with attention at baseline, and NfL with executive function at baseline (Supplementary Table 3). In the sensitivity analysis of CVSD, we observed similar trends, with the additional observation that the association of pTau181 with memory and attention over time also lost significance (Supplementary Table 4).

Nonetheless, across the entirety of the repeated sensitivity analyses, the majority of associations maintained consistent effect sizes and retained statistical significance.

Next, we analyzed each of the diagnostic groups separately (Table 2). In VCI, baseline associations between GFAP and NfL and cognitive performance largely remained similar to those in the total patient group, some with a marginally higher effect size. Associations between pTau181 and baseline cognitive performance in VCI remained largely similar in terms of effect sizes, but these results were no longer significant. As in the total group, lower Aβ_42/40_ ratio and higher GFAP and pTau181 were associated with cognitive decline over time in VCI (Table 2).

In HF, the baseline association between pTau181 and memory (β±SE –0.31±0.10; *q* < 0.01) remained, as did the baseline associations between GFAP and global cognition (β±SE –0.13±0.05; *q* < 0.05) and memory (β±SE –0.27±0.11; *q* < 0.05). NfL was not associated with baseline cognitive performance in HF. None of the biomarkers were associated with cognitive decline over time in HF.

In COD, a novel association between pTau181 and decline over time in executive functioning (β±SE –0.11±0.04; *q* < 0.05) appeared. None of the associations found in the total patient group remained in COD.

Baseline associations between pTau181, GFAP, and NfL remained most prominently present in patients with VCI and HF (Table 2).

## DISCUSSION

The main finding of this study is that plasma biomarkers for AD and related pathophysiological processes, such as astrocyte activation and axonal loss, are associated with cognitive impairment and cognitive decline over time in patients with cardiovascular diseases along the heart-brain axis, mostly attributable to patients with VCI and to a lesser extent in those with HF and COD.

In the whole cardiovascular patient group, higher GFAP and NfL were observed compared to the reference group. However, Aβ_42/40_ ratio and pTau181 were not different between the two groups. This implies that (co-morbid) AD pathology was limited in our current sample of patients with HF, COD, and VCI. Furthermore, more abnormal pTau181, GFAP, and NfL were associated with baseline cognitive impairment, and more abnormal Aβ_42/40_ ratio, pTau181, and GFAP concentrations were linked to a steeper decline in cognitive performance over time in multiple cognitive domains. The four blood-based biomarkers investigated in this study each reflect a different underlying aspect of AD-related pathology. Aβ_42/40_ ratio and pTau181 reflect the burden of Aβ deposition and tau pathology [11, 13]. As such, these markers, especially pTau181, may be viewed as most specific for AD pathology. In contrast, NfL is a less specific cross-disease biomarker of neurodegeneration, that is found in high concentrations in various neurological diseases [32, 33]. It is released due to axonal damage and reflects the degeneration of large myelinated axons [16, 17]. In addition, it has been proposed as biomarker for neurodegeneration related to CSVD burden [33, 34]. Notably, we found multiple associations between pTau181 and the rate of cognitive decline, while NfL was associated with baseline cognitive performance, but not with cognitive decline over time. These results might suggest that, while NfL is not fully specific for CSVD, AD pathology contributes to cognitive decline over time, while CSVD contributes to stable cognitive impairment. GFAP is a marker of astrocyte activation and has been considered as part of the neuroinflammatory response against amyloid-β plaques [14, 15]. A growing body of evidence suggests that plasma GFAP and amyloid burden are closely related [35, 36]. GFAP has also been related to CSVD, but only among those who were amyloid-positive, suggesting that astrocyte activation in CVSD may be specifically related to AD-related pathology [37]. The association between GFAP and cognitive decline over time suggests that cognitive decline in patients with cardiovascular diseases is not solely associated with amyloid and tau burden, as represented by pTau181, but also with neuro-inflammation.

When we stratified analysis by diagnostic group, we found that the majority of the cross-sectional and longitudinal associations between blood-based biomarkers and cognitive performance in patients with VCI were similar to those in the whole group of cardiovascular patients. Our findings are in line with prior evidence regarding the association between biomarkers for AD, as measured using CSF biomarkers or PET imaging, and cognition in VCI. Multiple studies suggested that Aβ binding (PET) and tau binding/concentrations (PET/CSF) are associated with cognitive decline over time in these patients [38–41]. Evidence based on blood-based biomarkers in relation to cognitive performance and cognitive decline in VCI is scarce. Two cross-sectional studies showed an association between serum Aβ_42_ or NfL and cognitive impairment in VCI [42, 43]. Our study extends on these findings by showing in a longitudinal design that plasma Aβ_42/40_ ratio, pTau181, and GFAP are not only associated with concurrent cognitive performance, but also with the rate of cognitive decline over time.

In patients with HF, the relationship between pTau181 and GFAP and baseline memory performance remained, while associations with cognitive decline over time lost significance. Our study may have been underpowered for the detection of a steeper rate of cognitive decline over time as patients remained stable on average. Although prior evidence is limited, one study conducted on patients with HF suggested that serum GFAP was also cross-sectionally associated with memory performance [44]. In addition, they found an association between serum GFAP and hippocampal atrophy. Thus, AD pathophysiology and astrocyte activation may play a role in cognitive impairment in patients with HF, but further research is needed to fully explore this association.

In patients with COD, none of the associations between blood-based biomarkers and cognitive performance remained, while a novel modest association between pTau181 and executive functioning appeared. Previous studies found that stenosis in the carotid artery was not associated with increased Aβ binding on PET scans, suggesting that COD is not associated with an increased production or impaired clearance of Aβ [45, 46]. Our results are in line with the notion that AD may not play a major role in cognitive decline in COD.

The mechanisms underpinning the relationship between AD and diseases along the heart-brain axis are not yet fully understood. There are several possible mechanisms by which these diseases may interact with AD. First, AD and cardiovascular diseases share multiple risk factors, including hypercholesterolemia, *APOE* status, diabetes mellitus, and obesity. Studies in animal models have shown that hypercholesteremia can increase the production of Aβ [47]. The *APOE* gene, which is involved in cholesterol metabolism, has also been linked to both cardiovascular diseases and AD, although the exact mechanism by which it affects these conditions is unclear [48]. Secondly, AD may be caused or exacerbated by the activation of the neurohormonal system, systemic inflammation, and microvascular dysfunction in the brain, all of which are common in cardiovascular diseases [49]. Thirdly, cerebral hypoperfusion caused by hemodynamic dysfunction in these diseases, may promote AD through multiple mechanisms, including the induction of oxidative stress, impaired clearance of Aβ, tau hyperphosphorylation, and neuroinflammation [50]. However, a previous study based on the Heart-Brain cohort did not find evidence for an association between cerebral hypoperfusion and cognitive performance in patients with disease along the heart-axis [7]. There are various factors that can influence the development and progression of both AD and cardiovascular diseases through different direct and indirect pathways that may act together in a synergistic manner.

Our study also has some potential limitations. In spite of this being one of the largest studies in its kind, patient groups were relatively small with a fairly short follow-up duration, which limited our ability to detect cognitive decline over time, especially in sub-group analyses. In addition, 30.9% of the participants were loss to follow up, due to death, illnesses unrelated to our study objectives, or nursing home admission. Patients with the most cognitive decline may not have been re-assessed, which could have caused underestimation of effect sizes.

Nonetheless, our study provides a comprehensive overview of the association between blood-based biomarkers for AD, astrocyte activation and neurodegeneration and cognitive performance in all major cardiovascular diseases along the heart-brain axis. Strengths of the current study are the inclusion of patients with various cardiovascular diseases along the heart-brain axis and the extensive neuropsychological assessment, covering all cognitive domains at baseline and follow-up. Our study highlights the potential of utilizing blood-based biomarkers, assessed through automated multiplex assays in plasma, to evaluate multiple aspects of AD pathophysiology and neurodegeneration in general. In the future, this approach might offer clinicians in cardiovascular disease an accessible tool to identify patients at risk for cognitive impairment or decline, facilitating cognitive evaluation or follow-up.

In summary, our results indicate that AD-related pathology, including astrocyte activation and neurodegeneration may contribute to cognitive impairment and cognitive decline over time in patients with cardiovascular diseases along the heart-brain axis, especially in patients with VCI and less pronounced in patients with HF and COD. Future research could explore these associations further by evaluating the interaction between AD-related, vascular specific and CVSD biomarkers on cognitive performance.

## AUTHOR CONTRIBUTIONS

Calvin Trieu (Formal analysis; Methodology; Software; Visualization; Writing – original draft; Writing – review & editing); Argonde C. van Harten (Conceptualization; Formal analysis; Investigation; Methodology; Supervision; Validation; Writing – review & editing); Anna E. Leeuwis (Conceptualization; Investigation; Methodology; Writing – review & editing); Lieza G. Exalto (Conceptualization; Investigation; Resources; Writing – review & editing); Astrid M. Hooghiemstra (Conceptualization; Data curation; Project administration; Writing – review & editing); Inge M. W. Verberk (Methodology; Writing – review & editing); Cor P. Allaart (Conceptualization; Funding acquisition; Investigation; Writing – review & editing); Hans-Peter Brunner-La Rocca (Conceptualization; Funding acquisition; Investigation; Writing – review & editing); L. Jaap Kappelle (Conceptualization; Funding acquisition; Investigation; Writing – review & editing); Robert J. van Oostenbrugge (Conceptualization; Funding acquisition; Investigation; Writing – review & editing); Geert-Jan Biessels (Conceptualization; Funding acquisition; Investigation; Writing – review & editing); Charlotte E. Teunissen (Conceptualization; Investigation; Methodology; Supervision; Writing – review & editing); Wiesje M. van der Flier (Conceptualization; Formal analysis; Funding acquisition; Investigation; Methodology; Supervision; Writing – review & editing).

## Supplementary Material

Supplementary Material

## Data Availability

Data may be shared (anonymized) for purposes of replicating procedures and results within the boundaries imposed by the informed consent and data sharing legislation.

## References

[1] Wolters FJ , Segufa RA , Darweesh SKL , Bos D , Ikram MA , Sabayan B , Hofman A , Sedaghat S (2018) Coronary heart disease, heart failure, and the risk of dementia: A systematic review and meta-analysis. Alzheimers Dement 14, 1493–1504. 29494808 10.1016/j.jalz.2018.01.007

[2] Liu Y , Braidy N , Poljak A , Chan DKY , Sachdev P (2018) Cerebral small vessel disease and the risk of Alzheimer’s disease: A systematic review. Ageing Res Rev 47, 41–48. 29898422 10.1016/j.arr.2018.06.002

[3] Gottesman RF , Albert MS , Alonso A , Coker LH , Coresh J , Davis SM , Deal JA , McKhann GM , Mosley TH , Sharrett AR , Schneider ALC , Windham BG , Wruck LM , Knopman DS (2017) Associations between midlife vascular risk factors and 25-year incident dementia in the Atherosclerosis Risk in Communities (ARIC) Cohort. JAMA Neurol 74, 1246–1254. 28783817 10.1001/jamaneurol.2017.1658 PMC5710244

[4] Santos CY , Snyder PJ , Wu W-C , Zhang M , Echeverria A , Alber J (2017) Pathophysiologic relationship between Alzheimer’s disease, cerebrovascular disease, and cardiovascular risk: A review and synthesis. Alzheimers Dement (Amst) 7, 69–87. 28275702 10.1016/j.dadm.2017.01.005 PMC5328683

[5] Hooghiemstra AM , Bertens AS , Leeuwis AE , Bron EE , Bots ML , Brunner-La Rocca HP , de Craen AJM , van der Geest RJ , Greving JP , Kappelle LJ , Niessen WJ , van Oostenbrugge RJ , van Osch MJP , de Roos A , van Rossum AC , Biessels GJ , van Buchem MA , Daemen M , van der Flier WM (2017) The missing link in the pathophysiology of vascular cognitive impairment: Design of the Heart-Brain Study. Cerebrovasc Dis Extra 7, 140–152. 29017156 10.1159/000480738 PMC5730112

[6] Hooghiemstra AM , Leeuwis AE , Bertens AS , Biessels GJ , Bots ML , Brunner-La Rocca HP , Greving JP , Kappelle LJ , van Oostenbrugge RJ , van Rossum AC , van der Flier WM (2019) Frequent cognitive impairment in patients with disorders along the heart-brain axis. Stroke 50, 3369–3375. 31684846 10.1161/STROKEAHA.119.026031

[7] Leeuwis AE , Hooghiemstra AM , Bron EE , Kuipers S , Oudeman EA , Kalay T , Brunner-La Rocca HP , Kappelle LJ , van Oostenbrugge RJ , Greving JP , Niessen WJ , van Buchem MA , van Osch MJP , van Rossum AC , Prins ND , Biessels GJ , Barkhof F , van der Flier WM (2020) Cerebral blood flow and cognitive functioning in patients with disorders along the heart-brain axis: Cerebral blood flow and the heart-brain axis. Alzheimers Dement (N Y) 6, e12034. 32995468 10.1002/trc2.12034 PMC7507476

[8] Scheltens P , De Strooper B , Kivipelto M , Holstege H , Chételat G , Teunissen CE , Cummings J , van der Flier WM (2021) Alzheimer’s disease. Lancet 397, 1577–1590. 33667416 10.1016/S0140-6736(20)32205-4 PMC8354300

[9] Teunissen CE , Verberk IMW , Thijssen EH , Vermunt L , Hansson O , Zetterberg H , van der Flier WM , Mielke MM , Del Campo M (2022) Blood-based biomarkers for Alzheimer’s disease: Towards clinical implementation. Lancet Neurol 21, 66–77. 34838239 10.1016/S1474-4422(21)00361-6

[10] Ashton NJ , Leuzy A , Lim YM , Troakes C , Hortobágyi T , Höglund K , Aarsland D , Lovestone S , Schöll M , Blennow K , Zetterberg H , Hye A (2019) Increased plasma neurofilament light chain concentration correlates with severity of post-mortem neurofibrillary tangle pathology and neurodegeneration. Acta Neuropathol Commun 7, 5. 10.1186/s40478-018-0649-3 PMC6327431 30626432

[11] Lantero Rodriguez J , Karikari TK , Suárez-Calvet M , Troakes C , King A , Emersic A , Aarsland D , Hye A , Zetterberg H , Blennow K , Ashton NJ (2020) Plasma p-tau181 accurately predicts Alzheimer’s disease pathology at least 8 years prior to post-mortem and improves the clinical characterisation of cognitive decline. Acta Neuropathol 140, 267–278. 32720099 10.1007/s00401-020-02195-x PMC7423866

[12] Winder Z , Sudduth TL , Anderson S , Patel E , Neltner J , Martin BJ , Snyder KE , Abner EL , Jicha GA , Nelson PT , Wilcock DM (2023) Examining the association between blood-based biomarkers and human post mortem neuropathology in the University of Kentucky Alzheimer’s Disease Research Center autopsy cohort. Alzheimers Dement 19, 67–78. 35266629 10.1002/alz.12639 PMC9463400

[13] Thijssen EH , La Joie R , Wolf A , Strom A , Wang P , Iaccarino L , Bourakova V , Cobigo Y , Heuer H , Spina S , VandeVrede L , Chai X , Proctor NK , Airey DC , Shcherbinin S , Duggan Evans C , Sims JR , Zetterberg H , Blennow K , Karydas AM , Teunissen CE , Kramer JH , Grinberg LT , Seeley WW , Rosen H , Boeve BF , Miller BL , Rabinovici GD , Dage JL , Rojas JC , Boxer AL (2020) Diagnostic value of plasma phosphorylated tau181 in Alzheimer’s disease and frontotemporal lobar degeneration. Nat Med 26, 387–397. 32123386 10.1038/s41591-020-0762-2 PMC7101073

[14] Pereira JB , Janelidze S , Smith R , Mattsson-Carlgren N , Palmqvist S , Teunissen CE , Zetterberg H , Stomrud E , Ashton NJ , Blennow K , Hansson O (2021) Plasma GFAP is an early marker of amyloid-β but not tau pathology in Alzheimer’s disease. Brain 144, 3505–3516. 34259835 10.1093/brain/awab223 PMC8677538

[15] Osborn LM , Kamphuis W , Wadman WJ , Hol EM (2016) Astrogliosis: An integral player in the pathogenesis of Alzheimer’s disease. Prog Neurobiol 144, 121–141. 26797041 10.1016/j.pneurobio.2016.01.001

[16] Gaiottino J , Norgren N , Dobson R , Topping J , Nissim A , Malaspina A , Bestwick JP , Monsch AU , Regeniter A , Lindberg RL , Kappos L , Leppert D , Petzold A , Giovannoni G , Kuhle J (2013) Increased neurofilament light chain blood levels in neurodegenerative neurological diseases. PLoS One 8, e75091. 24073237 10.1371/journal.pone.0075091 PMC3779219

[17] Schlaepfer WW , Lynch RG (1977) Immunofluorescence studies of neurofilaments in the rat and human peripheral and central nervous system. J Cell Biol 74, 241–250. 326799 10.1083/jcb.74.1.241 PMC2109861

[18] Ponikowski P , Voors AA , Anker SD , Bueno H , Cleland JGF , Coats AJS , Falk V , González-Juanatey JR , Harjola VP , Jankowska EA , Jessup M , Linde C , Nihoyannopoulos P , Parissis JT , Pieske B , Riley JP , Rosano GMC , Ruilope LM , Ruschitzka F , Rutten FH , van der Meer P (2016) 2016 ESC Guidelines for the diagnosis and treatment of acute and chronic heart failure: The Task Force for the diagnosis and treatment of acute and chronic heart failure of the European Society of Cardiology (ESC)Developed with the special contribution of the Heart Failure Association (HFA) of the ESC. Eur Heart J 37, 2129–2200. 27206819 10.1093/eurheartj/ehw128

[19] Folstein MF , Folstein SE , McHugh PR (1975) “Mini-mental state”. A practical method for grading the cognitive state of patients for the clinician. J Psychiatr Res 12, 189–198. 1202204 10.1016/0022-3956(75)90026-6

[20] Hughes CP , Berg L , Danziger WL , Coben LA , Martin RL (1982) A new clinical scale for the staging of dementia. Br J Psychiatry 140, 566–572. 7104545 10.1192/bjp.140.6.566

[21] Brink TL , Yesavage JA , Lum O , Heersema PH , Adey M , Rose TL (1982) Screening tests for geriatric depression. Clin Gerontol 1, 37–43. 10.1016/0022-3956(82)90033-4 7183759

[22] Staals J , Booth T , Morris Z , Bastin ME , Gow AJ , Corley J , Redmond P , Starr JM , Deary IJ , Wardlaw JM (2015) Total MRI load of cerebral small vessel disease and cognitive ability in older people. Neurobiol Aging 36, 2806–2811. 26189091 10.1016/j.neurobiolaging.2015.06.024 PMC4706154

[23] Aalten P , Ramakers IH , Biessels GJ , de Deyn PP , Koek HL , OldeRikkert MG , Oleksik AM , Richard E , Smits LL , van Swieten JC , Teune LK , van der Lugt A , Barkhof F , Teunissen CE , Rozendaal N , Verhey FR , van der Flier WM (2014) The Dutch Parelsnoer Institute – Neurodegenerative diseases; methods, design and baseline results. BMC Neurol 14, 254. 25551191 10.1186/s12883-014-0254-4 PMC4301568

[24] Van der Elst W , van Boxtel MP , van Breukelen GJ , Jolles J (2005) Rey’s verbal learning test: Normative data for 1855 healthy participants aged 24–81 years and the influence of age, sex, education, and mode of presentation. J Int Neuropsychol Soc 11, 290–302. 15892905 10.1017/S1355617705050344

[25] Lindeboom J , Schmand B , Tulner L , Walstra G , Jonker C (2002) Visual association test to detect early dementia of the Alzheimer type. J Neurol Neurosurg Psychiatry 73, 126–133. 12122168 10.1136/jnnp.73.2.126 PMC1737993

[26] Van der Elst W , Van Boxtel MP , Van Breukelen GJ , Jolles J (2006) Normative data for the Animal, Profession and Letter M Naming verbal fluency tests for Dutch speaking participants and the effects of age, education, and sex. J Int Neuropsychol Soc 12, 80–89. 16433947 10.1017/S1355617706060115

[27] Reitan RM (1955) The relation of the trail making test to organic brain damage. J Consult Psychol 19, 393–394. 13263471 10.1037/h0044509

[28] van der Elst W , van Boxtel MP , van Breukelen GJ , Jolles J (2006) The Letter Digit Substitution Test: Normative data for 1,858 healthy participants aged 24–81 from the Maastricht Aging Study (MAAS): Influence of age, education, and sex. J Clin Exp Neuropsychol 28, 998–1009. 16822738 10.1080/13803390591004428

[29] Van der Elst W , Van Boxtel MP , Van Breukelen GJ , Jolles J (2006) The Stroop color-word test: Influence of age, sex, and education; and normative data for a large sample across the adult age range. Assessment 13, 62–79. 16443719 10.1177/1073191105283427

[30] Lindeboom J , Matto D (1994) Digit series and Knox cubes as concentration tests for elderly subjects. Tijdschr Gerontol Geriatr 25, 63–68. 8197598

[31] Benjamini Y , Yekutieli D (2001) The control of the false discovery rate in multiple testing under dependency. Ann Statist 29, 1165–1188.

[32] Mattsson N , Insel PS , Palmqvist S , Portelius E , Zetterberg H , Weiner M , Blennow K , Hansson O (2016) Cerebrospinal fluid tau, neurogranin, and neurofilament light in Alzheimer’s disease. EMBO Mol Med 8, 1184–1196. 27534871 10.15252/emmm.201606540 PMC5048367

[33] Bridel C , van Wieringen WN , Zetterberg H , Tijms BM , Teunissen CE , Alvarez-Cermeño JC , Andreasson U , Axelsson M , Bäckström DC , Bartos A , Bjerke M , Blennow K , Boxer A , Brundin L , Burman J , Christensen T , Fialová L , Forsgren L , Frederiksen JL , Gisslén M , Gray E , Gunnarsson M , Hall S , Hansson O , Herbert MK , Jakobsson J , Jessen-Krut J , Janelidze S , Johannsson G , Jonsson M , Kappos L , Khademi M , Khalil M , Kuhle J , Landén M , Leinonen V , Logroscino G , Lu CH , Lycke J , Magdalinou NK , Malaspina A , Mattsson N , Meeter LH , Mehta SR , Modvig S , Olsson T , Paterson RW , Pérez-Santiago J , Piehl F , Pijnenburg YAL , Pyykkö OT , Ragnarsson O , Rojas JC , Romme Christensen J , Sandberg L , Scherling CS , Schott JM , Sellebjerg FT , Simone IL , Skillbäck T , Stilund M , Sundström P , Svenningsson A , Tortelli R , Tortorella C , Trentini A , Troiano M , Turner MR , van Swieten JC , Vågberg M , Verbeek MM , Villar LM , Visser PJ , Wallin A , Weiss A , Wikkelsø C , Wild EJ (2019) Diagnostic value of cerebrospinal fluid neurofilament light protein in neurology: A systematic review and meta-analysis. JAMA Neurol 76, 1035–1048. 31206160 10.1001/jamaneurol.2019.1534 PMC6580449

[34] Zhao Y , Xin Y , Meng S , He Z , Hu W (2019) Neurofilament light chain protein in neurodegenerative dementia: A systematic review and network meta-analysis. Neurosci Biobehav Rev 102, 123–138. 31026486 10.1016/j.neubiorev.2019.04.014

[35] Cicognola C , Janelidze S , Hertze J , Zetterberg H , Blennow K , Mattsson-Carlgren N , Hansson O (2021) Plasma glial fibrillary acidic protein detects Alzheimer pathology and predicts future conversion to Alzheimer dementia in patients with mild cognitive impairment. Alzheimers Res Ther 13, 68. 33773595 10.1186/s13195-021-00804-9 PMC8005231

[36] Verberk IMW , Thijssen E , Koelewijn J , Mauroo K , Vanbrabant J , de Wilde A , Zwan MD , Verfaillie SCJ , Ossenkoppele R , Barkhof F , van Berckel BNM , Scheltens P , van der Flier WM , Stoops E , Vanderstichele HM , Teunissen CE (2020) Combination of plasma amyloid beta((1–42/1–40)) and glial fibrillary acidic protein strongly associates with cerebral amyloid pathology. Alzheimers Res Ther 12, 118. 32988409 10.1186/s13195-020-00682-7 PMC7523295

[37] Shir D , Graff-Radford J , Hofrenning EI , Lesnick TG , Przybelski SA , Lowe VJ , Knopman DS , Petersen RC , Jack CR Jr. , Vemuri P , Algeciras-Schimnich A , Campbell MR , Stricker NH , Mielke MM (2022) Association of plasma glial fibrillary acidic protein (GFAP) with neuroimaging of Alzheimer’s disease and vascular pathology. Alzheimers Dement (Amst) 14, e12291. 35252538 10.1002/dad2.12291 PMC8883441

[38] Dao E , Best JR , Hsiung GR , Sossi V , Jacova C , Tam R , Liu-Ambrose T (2017) Associations between cerebral amyloid and changes in cognitive function and falls risk in subcortical ischemic vascular cognitive impairment. BMC Geriatr 17, 133. 28659161 10.1186/s12877-017-0522-4 PMC5490153

[39] Ye BS , Seo SW , Kim JH , Kim GH , Cho H , Noh Y , Kim HJ , Yoon CW , Woo SY , Kim SH , Park HK , Kim ST , Choe YS , Lee KH , Kim JS , Oh SJ , Kim C , Weiner M , Lee JH , Na DL (2015) Effects of amyloid and vascular markers on cognitive decline in subcortical vascular dementia. Neurology 85, 1687–1693. 26468407 10.1212/WNL.0000000000002097 PMC4653105

[40] Kim HJ , Park S , Cho H , Jang YK , San Lee J , Jang H , Kim Y , Kim KW , Ryu YH , Choi JY , Moon SH , Weiner MW , Jagust WJ , Rabinovici GD , DeCarli C , Lyoo CH , Na DL , Seo SW (2018) Assessment of extent and role of tau in subcortical vascular cognitive impairment using 18F-AV1451 positron emission tomography imaging. JAMA Neurol 75, 999–1007. 29799981 10.1001/jamaneurol.2018.0975 PMC6142932

[41] Jang H , Kim HJ , Choe YS , Kim SJ , Park S , Kim Y , Kim KW , Lyoo CH , Cho H , Ryu YH , Choi JY , DeCarli C , Na DL , Seo SW (2020) The impact of amyloid-β or tau on cognitive change in the presence of severe cerebrovascular disease. J Alzheimers Dis 78, 573–585. 33016911 10.3233/JAD-200680

[42] Ma W , Zhang J , Xu J , Feng D , Wang X , Zhang F (2020) Elevated levels of serum neurofilament light chain associated with cognitive impairment in vascular dementia. Dis Markers 2020, 6612871. 33204362 10.1155/2020/6612871 PMC7652600

[43] Uslu S , Akarkarasu ZE , Ozbabalik D , Ozkan S , Colak O , Demirkan ES , Ozkiris A , Demirustu C , Alatas O (2012) Levels of amyloid beta-42, interleukin-6 and tumor necrosis factor-alpha in Alzheimer’s disease and vascular dementia. Neurochem Res 37, 1554–1559. 22437436 10.1007/s11064-012-0750-0

[44] Traub J , Otto M , Sell R , Homola GA , Steinacker P , Oeckl P , Morbach C , Frantz S , Pham M , Störk S , Stoll G , Frey A (2022) Serum glial fibrillary acidic protein indicates memory impairment in patients with chronic heart failure. ESC Heart Fail 9, 2626–2634. 35611842 10.1002/ehf2.13986 PMC9288738

[45] Kang KM , Byun MS , Lee JH , Yi D , Choi HJ , Lee E , Lee Y , Lee JY , Kim YK , Sohn BK , Sohn CH , Lee DY (2020) Association of carotid and intracranial stenosis with Alzheimer’s disease biomarkers. Alzheimers Res Ther 12, 106. 32912336 10.1186/s13195-020-00675-6 PMC7488394

[46] Ogasawara K , Fujiwara S , Chida K , Terasaki K , Sasaki M , Kubo Y (2019) Reduction in amyloid β deposition on (18)F-florbetapir positron emission tomography with correction of cerebral hypoperfusion after endarterectomy for carotid stenosis. Am J Nucl Med Mol Imaging 9, 316–320. 31976161 PMC6971482

[47] Sparks DL , Martin TA , Gross DR , Hunsaker JC 3rd (2000) Link between heart disease, cholesterol, and Alzheimer’s disease: A review. Microsc Res Tech 50, 287–290. 10936882 10.1002/1097-0029(20000815)50:4<287::AID-JEMT7>3.0.CO;2-L

[48] Yang M , Li C , Zhang Y , Ren J (2020) Interrelationship between Alzheimer’s disease and cardiac dysfunction: The brain–heart continuum? Acta Biochim Biophys Sin 52, 1–8. 31897470 10.1093/abbs/gmz115

[49] Akiyama H , Barger S , Barnum S , Bradt B , Bauer J , Cole GM , Cooper NR , Eikelenboom P , Emmerling M , Fiebich BL , Finch CE , Frautschy S , Griffin WS , Hampel H , Hull M , Landreth G , Lue L , Mrak R , Mackenzie IR , McGeer PL , O’Banion MK , Pachter J , Pasinetti G , Plata-Salaman C , Rogers J , Rydel R , Shen Y , Streit W , Strohmeyer R , Tooyoma I , Van Muiswinkel FL , Veerhuis R , Walker D , Webster S , Wegrzyniak B , Wenk G , Wyss-Coray T (2000) Inflammation and Alzheimer’s disease. Neurobiol Aging 21, 383–421. 10858586 10.1016/s0197-4580(00)00124-x PMC3887148

[50] Zhao Y , Gong C-X (2015) From chronic cerebral hypoperfusion to Alzheimer-like brain pathology and neurodegeneration. Cell Mol Neurobiol 35, 101–110. 25352419 10.1007/s10571-014-0127-9 PMC11486181

