# Supplementary material for: Alzheimer’s Disease and Cognitive Decline in Patients with Cardiovascular Diseases Along the Heart-Brain Axis
Source: J Alzheimers Dis. 2024 Apr 2;98(3):987–1000. doi: 10.3233/JAD-231096 (PMC11091595; doi:10.3233/JAD-231096)
Supplement: Supplementary Material [file jad-98-jad231096-s001.pdf]

Supplementary Material

Alzheimer's Disease and Cognitive Decline in Patients with Cardiovascular Diseases Along the Heart-Brain Axis

Supplementary Table 1. Change in cognitive performance over time

	HF n = 153	COD n = 105	VCI n = 154	Reference participants n = 128	Total group n = 540
Estimated change over time					
Global cognition, β±SE	0.04±0.02*	0.02±0.02	-0.02±0.03	0.02±0.01	0.01±0.01
Memory, β±SE	0.10±0.04*	0.06±0.04	0.04±0.06	0.11±0.03**	0.08±0.03*
Attention/psychomotor speed, β±SE	0.00±0.02	-0.05±0.04	-0.05±0.04	0.02±0.01	-0.02±0.02
Language, β±SE	0.05±0.04	-0.01±0.02	-0.09±0.04*	-0.03±0.03	-0.02±0.02
Executive functioning, β±SE	0.01±0.03	0.08±0.03*	0.07±0.04	-0.01±0.02	0.03±0.02

Linear mixed model included terms for *time* in separate models for each cognitive domain. The estimates (β±SE) of βtime represent the annual change in cognitive performance. Analyses were adjusted for age, sex, hospital site, and education. *p < 0.05; **p < 0.001

Supplementary Table 2. Effect of blood-based biomarkers on baseline cognitive performance and cognitive performance over time (excluded patients fulfilling clinical AD Dementia criteria)

	**Patient groups**		**HF**		**COD**		**VCI**		**Reference participants**	
	Baseline z score β (SE)	Change over time β (SE)	Baseline z score β (SE)	Change over time β (SE)	Baseline z score β (SE)	Change over time β (SE)	Baseline z score β (SE)	Change over time β (SE)	Baseline z score β (SE)	Change over time β (SE)
$A\beta_{42/40}$ ratio										
GC	0.07 (0.04)	0.04 (0.01)*	0.04 (0.04)	-0.01 (0.02)	0.03 (0.06)	0.02 (0.02)	-0.04 (0.09)	0.07 (0.03)*	0.05 (0.04)	0.00 (0.01)
Memory	0.11 (0.08)	0.08 (0.04)*	0.12 (0.09)	-0.03 (0.04)	0.05 (0.10)	0.02 (0.05)	-0.29 (0.21)	0.19 (0.07)*	0.08 (0.05)	0.02 (0.02)
Attention	0.06 (0.06)	0.06 (0.02)*	0.04 (0.06)	0.00 (0.02)	0.02 (0.10)	0.05 (0.04)	-0.04 (0.13)	0.08 (0.04)	0.02 (0.06)	0.00 (0.01)
Language	0.07 (0.04)	0.01 (0.02)	0.04 (0.06)	0.01 (0.04)	-0.02 (0.06)	0.01 (0.03)	0.08 (0.06)	0.00 (0.03)	0.12 (0.06)	-0.03 (0.28)
EF	0.04 (0.04)	0.00 (0.02)	-0.05 (0.06)	-0.01 (0.03)	0.05 (0.07)	-0.01 (0.04)	0.09 (0.09)	0.02 (0.04)	0.00 (0.05)	0.02 (0.02)
pTau181										
GC	-0.07 (0.04)	-0.04 (0.02)	-0.09 (0.05)	-0.02 (0.02)	-0.02 (0.06)	0.00 (0.02)	-0.08 (0.08)	-0.08 (0.03)*	0.02 (0.04)	0.00 (0.01)
Memory	-0.18 (0.09)	-0.04 (0.04)	-0.31 (0.10)*	0.03 (0.05)	-0.07 (0.11)	0.09 (0.05)	-0.11 (0.18)	-0.18 (0.07)*	0.03 (0.07)	0.01 (0.03)
Attention	-0.05 (0.06)	-0.03 (0.03)	-0.07 (0.07)	0.00 (0.03)	0.01 (0.11)	-0.01 (0.05)	-0.10 (0.12)	-0.09 (0.04)*	-0.03 (0.07)	0.01 (0.02)
Language	0.00 (0.04)	-0.03 (0.02)	0.06 (0.07)	-0.05 (0.05)	-0.02 (0.06)	0.02 (0.03)	-0.02 (0.06)	-0.03 (0.03)	0.04 (0.08)	-0.03 (0.03)
EF	-0.04 (0.04)	-0.04 (0.02)	-0.04 (0.07)	-0.04 (0.04)	0.00 (0.08)	-0.11 (0.04)*	-0.07 (0.08)	-0.01 (0.03)	0.03 (0.06)	0.02 (0.02)
GFAP										
GC	-0.16 (0.05)**	-0.03 (0.01)	-0.13 (0.05)*	-0.01 (0.02)	0.00 (0.00)	-0.01 (0.02)	-0.19 (0.09)	-0.05 (0.03)	-0.01 (0.05)	0.00 (0.01)
Memory	-0.26 (0.10)*	-0.01 (0.04)	-0.27 (0.11)*	0.01 (0.04)	0.02 (0.13)	0.00 (0.05)	-0.25 (0.19)	-0.05 (0.07)	-0.07 (0.08)	0.02 (0.03)
Attention	-0.12 (0.07)	-0.02 (0.02)	-0.07 (0.07)	0.00 (0.03)	0.04 (0.13)	0.01 (0.04)	-0.18 (0.13)	-0.08 (0.04)	-0.01 (0.08)	-0.02 (0.02)
Language	-0.12 (0.04)*	-0.02 (0.02)	-0.06 (0.07)	-0.04 (0.04)	-0.04 (0.07)	-0.01 (0.03)	-0.14 (0.06)	-0.01 (0.03)	-0.01 (0.09)	-0.02 (0.03)
EF	-0.12 (0.05)*	-0.03 (0.02)	-0.12 (0.07)	-0.02 (0.03)	-0.01 (0.09)	-0.04 (0.04)	-0.16 (0.09)	-0.04 (0.03)	0.03 (0.07)	-0.01 (0.02)
NfL										
GC	-0.11 (0.04)*	-0.02 (0.01)	-0.08 (0.06)	-0.02 (0.02)	0.03 (0.05)	0.01 (0.02)	-0.26 (0.08)**	-0.04 (0.02)	0.00 (0.07)	0.01 (0.02)
Memory	-0.17 (0.09)	-0.03 (0.04)	-0.14 (0.13)	-0.05 (0.05)	0.03 (0.09)	0.00 (0.04)	-0.37 (0.17)*	-0.04 (0.06)	0.15 (0.10)	0.04 (0.04)
Attention	-0.18 (0.06)*	-0.01 (0.02)	-0.18 (0.08)	0.00 (0.03)	0.01 (0.09)	0.03 (0.04)	-0.34 (0.11)*	-0.06 (0.04)	-0.09 (0.10)	-0.03 (0.02)
Language	-0.05 (0.04)	-0.03 (0.02)	0.10 (0.09)	0.01 (0.05)	0.05 (0.05)	-0.01 (0.02)	-0.18 (0.05)**	-0.05 (0.03)	0.06 (0.12)	0.02 (0.05)
EF	-0.07 (0.04)	0.00 (0.02)	-0.09 (0.08)	0.00 (0.04)	0.03 (0.06)	0.02 (0.03)	-0.13 (0.08)	-0.01 (0.03)	-0.10 (0.09)	0.00 (0.03)

EF, Executive functioning; GC, Global cognition

Linear mixed model included terms for *biomarker*, *time*, and *biomarker*time interaction* in separate models for each biomarker and each cognitive domain. Analyses were adjusted for age, sex, hospital site and education. The estimates (β (SE)) with the term for *biomarker* (baseline z score) represent the association between the blood-based biomarker and baseline cognitive performance. Estimates with the term for *biomarker*time interaction* (change over time) represent the association between the blood-based biomarkers and annual change in cognitive performance over time. For $A\beta_{42/40}$ ratio positive estimates indicate that lower concentrations of this biomarker are associated with worse cognitive performance. In contrast, for pTau181, GFAP, and NFL, negative estimates indicate that higher concentrations of these biomarkers are associated with worse cognitive performance. The p-values are FDR-corrected and represent a significant association between the blood-based biomarker and cognitive performance of the designated cognitive domain at baseline (baseline z score) or over time (change over time). *$q_{FDR} < 0.05$; **$q_{FDR} < 0.001$

Supplementary Table 3. Effect of blood-based biomarkers on baseline cognitive performance and cognitive performance over time (adjusted for age, sex, hospital site, education and GDS)

	**Patient groups**		**HF**		**COD**		**VCI**		**Reference participants**	
	Baseline z score β (SE)	Change over time β (SE)	Baseline z score β (SE)	Change over time β (SE)	Baseline z score β (SE)	Change over time β (SE)	Baseline z score β (SE)	Change over time β (SE)	Baseline z score β (SE)	Change over time β (SE)
$A\beta_{42/40}$ ratio										
GC	0.07 (0.04)	0.03 (0.02)	0.04 (0.04)	-0.01 (0.02)	0.04 (0.05)	0.02 (0.02)	-0.06 (0.10)	0.10 (0.03)*	0.05 (0.04)	0.00 (0.01)
Memory	0.13 (0.09)	0.07 (0.04)	0.12 (0.08)	-0.04 (0.04)	0.06 (0.11)	0.02 (0.05)	-0.26 (0.21)	0.22 (0.07)*	0.07 (0.05)	0.02 (0.02)
Attention	0.05 (0.05)	0.05 (0.02)	0.04 (0.06)	0.00 (0.02)	0.03 (0.10)	0.05 (0.04)	-0.06 (0.12)	0.11 (0.04)*	0.01 (0.06)	0.00 (0.01)
Language	0.06 (0.04)	0.02 (0.02)	0.04 (0.06)	0.01 (0.04)	0.00 (0.06)	0.01 (0.03)	0.02 (0.08)	0.02 (0.04)	0.11 (0.06)	-0.03 (0.03)
EF	0.04 (0.04)	0.01 (0.02)	-0.05 (0.06)	-0.01 (0.03)	0.07 (0.07)	-0.01 (0.04)	0.06 (0.09)	0.04 (0.04)	0.00 (0.05)	0.02 (0.02)
pTau181										
GC	-0.10 (0.04)*	-0.06 (0.02)**	-0.07 (0.05)	-0.02 (0.02)	-0.03 (0.06)	0.00 (0.02)	-0.11 (0.09)	-0.13 (0.03)**	0.02 (0.04)	0.00 (0.01)
Memory	-0.25 (0.09)*	-0.09 (0.04)*	-0.27 (0.09)*	0.03 (0.05)	-0.06 (0.11)	0.09 (0.05)	-0.16 (0.19)	-0.27 (0.07)**	0.03 (0.07)	0.01 (0.03)
Attention	-0.07 (0.06)	-0.07 (0.02)*	-0.05 (0.07)	0.00 (0.03)	-0.03 (0.11)	-0.01 (0.05)	-0.10 (0.11)	-0.16 (0.04)**	-0.03 (0.07)	0.01 (0.02)
Language	-0.02 (0.04)	-0.06 (0.02)*	0.07 (0.07)	-0.05 (0.05)	-0.05 (0.06)	0.02 (0.03)	-0.05 (0.08)	-0.09 (0.04)*	0.05 (0.07)	-0.03 (0.03)
EF	-0.06 (0.05)	-0.03 (0.02)	-0.03 (0.07)	-0.04 (0.04)	0.01 (0.08)	-0.10 (0.04)	-0.12 (0.09)	0.02 (0.04)	0.03 (0.06)	0.02 (0.02)
GFAP										
GC	-0.18 (0.05)**	-0.05 (0.01)*	-0.13 (0.05)*	-0.01 (0.02)	-0.01 (0.07)	-0.01 (0.02)	-0.20 (0.09)	-0.09 (0.03)*	0.00 (0.05)	0.00 (0.01)
Memory	-0.33 (0.10)**	-0.05 (0.03)	-0.27 (0.10)*	0.02 (0.04)	0.02 (0.13)	0.00 (0.05)	-0.36 (0.20)	-0.13 (0.06)	-0.06 (0.08)	0.02 (0.03)
Attention	-0.11 (0.06)	-0.05 (0.02)*	-0.07 (0.07)	0.00 (0.03)	0.01 (0.12)	0.01 (0.04)	-0.13 (0.11)	-0.13 (0.04)**	0.00 (0.08)	-0.02 (0.02)
Language	-0.12 (0.04)*	-0.04 (0.02)	-0.06 (0.07)	-0.04 (0.04)	-0.06 (0.07)	0.00 (0.03)	-0.13 (0.04)	-0.07 (0.03)	0.01 (0.09)	-0.02 (0.03)
EF	-0.14 (0.05)*	-0.01 (0.02)	-0.12 (0.07)	-0.02 (0.03)	-0.02 (0.09)	-0.04 (0.04)	-0.17 (0.09)	-0.01 (0.04)	0.05 (0.07)	-0.01 (0.02)
NfL										
GC	-0.12 (0.04)*	-0.03 (0.01)	-0.06 (0.06)	-0.02 (0.02)	0.03 (0.05)	0.01 (0.02)	-0.27 (0.09)*	-0.05 (0.03)	-0.02 (0.07)	0.01 (0.02)
Memory	-0.21 (0.09)*	-0.06 (0.03)	-0.08 (0.12)	-0.05 (0.05)	0.04 (0.09)	0.00 (0.04)	-0.48 (0.19)*	-0.07 (0.06)	0.13 (0.10)	0.04 (0.04)
Attention	-0.16 (0.06)*	-0.03 (0.02)	-0.16 (0.08)	-0.00 (0.03)	0.01 (0.09)	0.03 (0.04)	-0.27 (0.11)*	-0.09 (0.04)	-0.10(0.11)	-0.03 (0.02)
Language	-0.03 (0.04)	-0.03 (0.02)	0.11 (0.09)	0.01 (0.05)	0.05 (0.05)	-0.01 (0.02)	-0.16 (0.07)*	-0.05 (0.03)	0.03 (0.11)	0.02 (0.05)
EF	-0.07 (0.04)	0.01 (0.02)	-0.08 (0.08)	0.00 (0.04)	0.02 (0.06)	0.02 (0.03)	-0.15 (0.08)	0.03 (0.04)	-0.13 (0.09)	0.00 (0.03)

EF, Executive functioning; GC, Global cognition; GDS, Geriatric Depression Scale

Linear mixed model included terms for *biomarker*, *time*, and *biomarker*time interaction* in separate models for each biomarker and each cognitive domain. Analyses were adjusted for age, sex, hospital site, education, and GDS. The estimates (β (SE)) with the term for *biomarker* (baseline z score) represent the association between the blood-based biomarker and baseline cognitive performance. Estimates with the term for *biomarker*time interaction* (change over time) represent the association between the blood-based biomarkers and annual change in cognitive performance over time. For $A\beta_{42/40}$ ratio positive estimates indicate that lower concentrations of this biomarker are associated with worse cognitive performance. In contrast, for pTau181, GFAP and NFL, negative estimates indicate that higher concentrations of these biomarkers are associated with worse cognitive performance. The p-values are FDR-corrected and represent a significant association between the blood-based biomarker and cognitive performance of the designated cognitive domain at baseline (baseline z score) or over time (change over time). $^*q_{FDR} < 0.05$; $^{**}q_{FDR} < 0.001$

Supplementary Table 4. Effect of blood-based biomarkers on baseline cognitive performance and cognitive performance over time (adjusted for age, sex, hospital site, education and CSVD score)

	**Patient groups**		**HF**		**COD**		**VCI**		**Reference participants**	
	Baseline z score β (SE)	Change over time β (SE)	Baseline z score β (SE)	Change over time β (SE)	Baseline z score β (SE)	Change over time β (SE)	Baseline z score β (SE)	Change over time β (SE)	Baseline z score β (SE)	Change over time β (SE)
$A\beta_{42/40}$ ratio										
GC	0.08 (0.05)	0.04 (0.02)	0.02 (0.04)	-0.01 (0.02)	0.02 (0.06)	0.01 (0.02)	-0.02 (0.11)	0.11 (0.03)**	0.05 (0.04)	0.00 (0.01)
Memory	0.10 (0.10)	0.07 (0.04)	0.05 (0.09)	-0.04 (0.04)	0.02 (0.11)	0.03 (0.05)	-0.27 (0.23)	0.21 (0.07)*	0.08 (0.06)	0.02 (0.02)
Attention	0.05 (0.06)	0.03 (0.02)	0.01 (0.06)	0.00 (0.03)	0.04 (0.11)	0.04 (0.04)	0.02 (0.13)	0.06 (0.04)	0.01 (0.06)	0.00 (0.01)
Language	0.11 (0.05)	0.05 (0.03)	0.05 (0.06)	0.00 (0.04)	0.02 (0.06)	0.01 (0.03)	0.09 (0.11)	0.10 (0.05)	0.12 (0.06)	-0.03 (0.03)
EF	0.06 (0.04)	0.01 (0.02)	-0.02 (0.06)	0.00 (0.03)	0.05 (0.08)	-0.02 (0.04)	0.09 (0.09)	0.05 (0.04)	0.00 (0.05)	0.02 (0.02)
pTau181										
GC	-0.15 (0.05)*	-0.07 (0.02)**	-0.11 (0.05)	-0.03 (0.02)	0.02 (0.07)	0.01 (0.02)	-0.20 (0.10)	-0.14 (0.03)**	0.01 (0.05)	0.00 (0.01)
Memory	-0.35 (0.10)**	-0.08 (0.04)	-0.35 (0.10)**	0.03 (0.05)	-0.04 (0.13)	0.10 (0.05)	-0.35 (0.21)	-0.24 (0.07)**	0.03 (0.07)	0.01 (0.03)
Attention	-0.10 (0.06)	-0.05 (0.02)	-0.06 (0.07)	0.00 (0.03)	0.02 (0.13)	0.00 (0.05)	-0.16 (0.12)	-0.12 (0.04)*	-0.02 (0.07)	0.01 (0.02)
Language	-0.08 (0.05)	-0.11 (0.03)*	0.04 (0.07)	-0.07 (0.05)	0.02 (0.07)	0.01 (0.03)	-0.16 (0.10)	-0.17 (0.05)**	0.04 (0.08)	-0.03 (0.03)
EF	-0.07 (0.05)	-0.04 (0.02)	-0.07 (0.07)	-0.05 (0.04)	0.05 (0.09)	-0.10 (0.04)	-0.12 (0.09)	0.00 (0.04)	0.02 (0.06)	0.02 (0.02)
GFAP										
GC	-0.22 (0.05)**	-0.05 (0.02)*	-0.11 (0.05)	-0.02 (0.02)	0.03 (0.08)	-0.02 (0.02)	-0.28 (0.10)*	-0.09 (0.03)*	-0.01 (0.05)	0.00 (0.01)
Memory	-0.49 (0.11)**	-0.04 (0.04)	-0.29 (0.11)*	0.02 (0.05)	0.09 (0.15)	0.00 (0.05)	-0.62 (0.22)*	-0.10 (0.07)	-0.07 (0.08)	0.02 (0.03)
Attention	-0.11 (0.07)	-0.05 (0.02)	0.00 (0.08)	0.00 (0.03)	0.07 (0.15)	0.01 (0.04)	-0.17 (0.12)	-0.11 (0.04)*	0.020(0.08)	-0.02 (0.02)
Language	-0.14 (0.06)*	-0.06 (0.03)*	-0.06 (0.08)	-0.04 (0.05)	-0.01 (0.08)	-0.02 (0.03)	-0.15 (0.11)	-0.09 (0.04)	-0.01 (0.09)	-0.02 (0.04)
EF	-0.13 (0.05)*	-0.02 (0.02)	-0.10 (0.07)	-0.01 (0.03)	0.02 (0.10)	-0.04 (0.04)	-0.17 (0.09)	-0.02 (0.04)	0.03 (0.07)	-0.01 (0.02)
NfL										
GC	-0.17 (0.05)**	-0.03 (0.02)	-0.13 (0.06)	-0.03 (0.02)	0.05 (0.06)	0.00 (0.02)	-0.32 (0.10)*	-0.04 (0.03)	0.01 (0.07)	0.01 (0.02)
Memory	-0.35 (0.10)**	-0.03 (0.04)	-0.21 (0.13)	-0.06 (0.05)	0.04 (0.11)	0.00 (0.05)	-0.68 (0.21)*	-0.01 (0.07)	0.16 (0.10)	0.04 (0.04)
Attention	-0.19 (0.06)*	-0.02 (0.02)	-0.18 (0.09)	0.00 (0.03)	0.06 (0.11)	0.03 (0.04)	-0.30 (0.12)*	-0.06 (0.04)	-0.07 (0.11)	-0.03 (0.02)
Language	-0.07 (0.06)	-0.05 (0.03)	0.05 (0.09)	0.00 (0.05)	0.08 (0.06)	-0.03 (0.03)	-0.17 (0.10)	-0.08 (0.05)	0.05 (0.12)	0.02 (0.05)
EF	-0.08 (0.05)	-0.01 (0.02)	-0.16 (0.09)	-0.02 (0.04)	0.01 (0.08)	0.00 (0.04)	-0.13 (0.09)	0.00 (0.04)	-0.09 (0.09)	0.00 (0.03)

CSVD, Cerebral small vessel disease; EF, Executive functioning; GC, Global cognition

Linear mixed model included terms for *biomarker*, *time*, and *biomarker*time interaction* in separate models for each biomarker and each cognitive domain. Analyses were adjusted for age, sex, hospital site, education, and CSVD score. The estimates (β (SE)) with the term for *biomarker* (baseline z score) represent the association between the blood-based biomarker and baseline cognitive performance. Estimates with the term for *biomarker*time interaction* (change over time) represent the association between the blood-based biomarkers and annual change in cognitive performance over time. For $A\beta_{42/40}$ ratio positive estimates indicate that lower concentrations of this biomarker are associated with worse cognitive performance. In contrast, for pTau181, GFAP and NFL, negative estimates indicate that higher concentrations of these biomarkers are associated with worse cognitive performance. The p-values are FDR-corrected and represent a significant association between the blood-based biomarker and cognitive performance of the designated cognitive domain at baseline (baseline z score) or over time (change over time). $^*q_{FDR} < 0.05$; $^{**}q_{FDR} < 0.001$

STROBE Statement—Checklist of items that should be included in reports of ***cohort studies***

	Item No	Recommendation	Page No
**Title and abstract**	1	(*a*) Indicate the study's design with a commonly used term in the title or the abstract<br>(*b*) Provide in the abstract an informative and balanced summary of what was done and what was found	1-3
**Introduction**			
Background/rationale	2	Explain the scientific background and rationale for the investigation being reported	4-5
Objectives	3	State specific objectives, including any prespecified hypotheses	5
**Methods**			
Study design	4	Present key elements of study design early in the paper	6
Setting	5	Describe the setting, locations, and relevant dates, including periods of recruitment, exposure, follow-up, and data collection	6&8
Participants	6	(*a*) Give the eligibility criteria, and the sources and methods of selection of participants. Describe methods of follow-up<br>(*b*) For matched studies, give matching criteria and number of exposed and unexposed	6&8
Variables	7	Clearly define all outcomes, exposures, predictors, potential confounders, and effect modifiers. Give diagnostic criteria, if applicable	6-8
Data sources/ measurement	8*	For each variable of interest, give sources of data and details of methods of assessment (measurement). Describe comparability of assessment methods if there is more than one group	7-9
Bias	9	Describe any efforts to address potential sources of bias	9-10
Study size	10	Explain how the study size was arrived at	NA
Quantitative variables	11	Explain how quantitative variables were handled in the analyses. If applicable, describe which groupings were chosen and why	9-10
Statistical methods	12	(*a*) Describe all statistical methods, including those used to control for confounding<br>(*b*) Describe any methods used to examine subgroups and interactions<br>(*c*) Explain how missing data were addressed<br>(*d*) If applicable, explain how loss to follow-up was addressed<br>(*e*) Describe any sensitivity analyses	9-10
**Results**			
Participants	13*	(a) Report numbers of individuals at each stage of study—e.g., numbers potentially eligible, examined for eligibility, confirmed eligible, included in the study, completing follow-up, and analyzed<br>(b) Give reasons for non-participation at each stage<br>(c) Consider use of a flow diagram	6&11
Descriptive data	14*	(a) Give characteristics of study participants (e.g., demographic, clinical, social) and information on exposures and potential confounders<br>(b) Indicate number of participants with missing data for each variable of interest<br>(c) Summarize follow-up time (e.g., average and total amount)	11
Outcome data	15*	Report numbers of outcome events or summary measures over time	11-13

Main results	16	(*a*) Give unadjusted estimates and, if applicable, confounder-adjusted estimates and their precision (e.g., 95% confidence interval). Make clear which confounders were adjusted for and why they were included<br>(*b*) Report category boundaries when continuous variables were categorized<br>(*c*) If relevant, consider translating estimates of relative risk into absolute risk for a meaningful time period	11-13
Other analyses	17	Report other analyses done—e.g., analyses of subgroups and interactions, and sensitivity analyses	12-13
**Discussion**			
Key results	18	Summarize key results with reference to study objectives	14
Limitations	19	Discuss limitations of the study, taking into account sources of potential bias or imprecision. Discuss both direction and magnitude of any potential bias	17
Interpretation	20	Give a cautious overall interpretation of results considering objectives, limitations, multiplicity of analyses, results from similar studies, and other relevant evidence	17
Generalizability	21	Discuss the generalizability (external validity) of the study results	17
**Other information**			
Funding	22	Give the source of funding and the role of the funders for the present study and, if applicable, for the original study on which the present article is based	18

*Give information separately for exposed and unexposed groups.

Note: An Explanation and Elaboration article discusses each checklist item and gives methodological background and published examples of transparent reporting. The STROBE checklist is best used in conjunction with this article (freely available on the Web sites of PLoS Medicine at http://www.plosmedicine.org/, Annals of Internal Medicine at http://www.annals.org/, and Epidemiology at http://www.epidem.com/). Information on the STROBE Initiative is available at http://www.strobe-statement.org.
